# Long-term retrospective assessment of a transmission hotspot for human alveolar echinococcosis in mid-west China

**DOI:** 10.1371/journal.pntd.0007701

**Published:** 2019-08-30

**Authors:** Patrick Giraudoux, YuMin Zhao, Eve Afonso, HongBin Yan, Jenny Knapp, Michael T. Rogan, DaZhong Shi, WanZhong Jia, Philip S. Craig

**Affiliations:** 1 Chrono-environment, UMR UFC/CNRS 6249 aff. INRA, Université de Franche-Comté, Besançon, France; 2 Laboratory of Wildlife Management and Ecosystem Health, Yunnan University of Finance and Economics, Kunming, China; 3 School of Basic Medicine, Guilin Medical University, Guilin, Guangxi, China; 4 Institute of Pathogenic Biology, Lanzhou University, Lanzhou, Gansu, China; 5 Lanzhou Veterinary Research Institute, Chinese Academy of Agricultural Sciences, Lanzhou, Gansu, China; 6 School of Environment and Life Sciences, University of Salford, Greater Manchester, United Kingdom; IRNASA, CSIC, SPAIN

## Abstract

**Background:**

Human alveolar echinococcosis caused by infection with *Echinococcus multilocularis* is one of the most potentially pathogenic helminthic zoonoses. Transmission occurs involving wildlife cycles typically between fox and small mammal intermediate hosts. In the late 1980s/early 1990s a large focus of human AE was identified in poor upland agricultural communities in south Gansu Province, China. More detailed investigations in 1994–97 expanded community screening and identified key risk factors of dog ownership and landscape type around villages that could support susceptible rodent populations. A crash of the dog population (susceptible domestic definitive host) in the early 1990s appeared to stop transmission.

**Methodology/Findings:**

We subsequently undertook follow-up eco-epidemiological studies based on human population screening and dog survey, in 2005/6 and in 2014/15. Our observations show a decrease in human AE prevalence, especially marked in the 11–30 year old age category. In 2015, although the dog population had recovered and in addition, forest protection and the reforestation of some areas may have favoured red fox (wild definitive host) population growth, there was no evidence of infection in owned dogs.

**Conclusions/Significance:**

Those observations suggest that over decades socio-ecological changes resulted in a cascade of factors that exacerbated and then interrupted parasite emergence, with probable elimination of peri-domestic transmission of *E*. *multilocularis* in this area, despite the relative proximity of large active transmission foci on the eastern Tibetan Plateau. This study case exemplifies how anthropogenic land use and behavioural changes can modify emergence events and the transmission of endemic zoonotic parasite infections, and subsequently the importance of considering processes over the long-term in a systems approach in order to understand pathogen and disease distribution.

## Author summary

Human alveolar echinococcosis caused by infection with *Echinococcus multilocularis* is one of the most potentially pathogenic helminthic zoonoses. Transmission occurs involving wildlife cycles typically between fox and small mammal intermediate hosts. A large focus of human alveolar echinococcosis was identified in the late 1980s in poor upland agricultural communities in south Gansu Province, China, and has been monitored until 2015. Observations suggest that over decades landscape and socio-ecological changes resulted in a cascade of factors that exacerbated and then interrupted parasite emergence, with probable elimination of peri-domestic transmission of *E*. *multilocularis* in this area, despite the relative proximity of large active transmission foci on the eastern Tibetan Plateau. This study case exemplifies how anthropogenic land use and behavioural changes can modify emergence events and the long-term transmission of endemic parasitic infections, and subsequently the importance of considering disease ecology transmission socio-ecosystems in order to understand parasite and disease distribution.

## Introduction

Zoonotic infections that involve domesticated animals and/or wildlife hosts are of increasing concern globally, especially in resource-poor rural communities, and they are usually difficult to monitor, treat and control [1]. Chronic zoonotic parasitic helminthic infections such as trematodiases, cysticercosis and echinococcosis pose additional difficulties caused by long-term pathologies and variable periods of asymptomatology followed by non-specific symptoms. Human alveolar echinococcosis (AE) results from accidental infection with eggs of the canid small tapeworm *Echinococcus multilocularis*. It is one of the most pathogenic helminthic infections due to development of hepatic multivesiculated metacestode lesions with tissue fibrosis, necrosis and metastatic potential. The life cycle of *E*. *multilocularis* involves carnivores as definitive hosts (primarily foxes but also dogs) and small mammal herbivores such as rodents and lagomorphs as intermediate hosts [2]. Despite being globally rare, human AE places a serious burden on affected communities in focal endemic areas and remains difficult and expensive to diagnose and treat. As for many zoonoses, incidence rates of human AE are associated with lifestyle, host ecology and specific transmission ecosystems [3–10]. For example Tibetan pastoral communities of alpine valleys in northwest Sichuan are estimated to lose 0.81 Disability Adjusted Life Years (DALYs) per person due to alveolar and cystic echinococcosis, compared to an average 0.18 DALYs lost in the general Chinese population due to all communicable and non-communicable ailments combined [11]. It is now clear that echinococcosis is a major burden for communities in endemic areas of China and poses a public health problem of primary importance [12].

Since the first hydatid control programme for cystic echinococcosis was implemented in Iceland in the 1860s [13], at least 20 intervention programmes have been undertaken in different world regions targeting mostly *E*. *granulosus* and less frequently transmission of *E*. *multilocularis*. The first control programme for AE occurred in Reubun Island, Japan, from the 1940s when the fox population was eliminated [14]. Modern control of *E*. *multilocularis* transmission can no longer rely on fox elimination considering the large scale at which such programmes should be implemented on continents and the subsequent ethical, ecological and technical issues raised by such targets [15]. However distribution of baits containing praziquantel can have significant impacts on vulpine prevalence of *E*. *multilocularis*, but is difficult to maintain over long periods and large geographic areas [16]. Contact with dog definitive hosts can be a major risk factor for human AE infection in communities where dogs live in close vicinity to humans, for example on St Lawrence Island in the Bering sea [17] and rural areas of China [5,18].

A critical aspect of echinococcosis control is the definition of an adequate baseline at the beginning of the intervention and the appropriate surveillance of disease incidence or prevalence in well targeted hosts [14]. Metacestode development in human AE is extremely slow with an asymptomatic period of 5 to 15 years or more [19,20]. A consequence is that the epidemiology of human AE at a given time in a given area might not reflect the current transmission status in the area, but, with a time lag, integrate over years the results of transmission systems that have been variously active decades ago. This is the case when environmental conditions conducive to transmission have changed naturally or as a result of anthropogenic impacts. This is further complicated when chronic diseases, such as AE, often fail to cause hospitalization or hospital records are inadequate, thus the `memory`of the epidemiological baseline can be lost. In ecology, quantified long-term adaptive monitoring [21], although crucial is still often problematic (short-term funding and difficult metrology of wildlife population variables, etc.) [22].

In the absence of a long-term adaptive monitoring framework, *post-hoc* ‘reactive’ monitoring can be implemented [23]. It is based on collecting available historical data and assembling them in a retro-observatory database in order to rebuild the time series that should have been monitored longitudinally. This can be a way for documenting environmental and epidemiological changes and understanding how transmission patterns can evolve over the long term and help to design more sustainable integrated control strategies and better prevent risks of re-emergence after parasite elimination.

The first comprehensive early studies on human AE and the parasite transmission ecology in China were undertaken in the early 1990s in Han agricultural communities in southern Gansu Province. Prior to that, in the 1980s, a cluster of AE cases were reported from hospitals in Lanzhou (capital city of Gansu) that originated from Zhang county, a poor partly terraced upland region about 250km to the south [24]. A subsequent investigation (in 1991) of six communities in two valleys in Zhang county revealed initially a high seroprevalence (8.8%) for specific *E*. *multilocularis* antibodies in a sample population (n = 606). Following that sero-survey confirmation of AE lesions in seropositives was undertaken and a community mass screening program was made using hepatic ultrasound scanning in a larger population (n = 1312). That study indicated a 5% prevalence of hepatic AE with a case age range of 11–73 years and a mean of 40.9 years [25]. Furthermore a necropsy study of unwanted dogs revealed a 10% prevalence of intestinal *E*. *multilocularis* infection, however the local red fox *(Vulpes vulpes)* population was not examined, neither were small mammals, though field mice (*Apodemus* spp.) and zokors (*Eospalax* spp.) were commonly trapped by locals close to villages [25].

Three years later, between 1994 and 1997, a much larger study was undertaken in Zhang county and the neighbouring Puma district (Min county), in which 2482 persons were voluntarily screened in 1994–6 by portable ultrasound in their villages (n = 31). That program detected 84 AE cases (3.4% prevalence, but increasing to 4.1% when AE cases identified in the 1991 survey were included) with a mean age of 38.7 years (range 12–70 years) [18]. Village human AE prevalences varied from 0% to >10% and 9 villages had AE prevalences >5%, with main risk factors for an AE case being: female >20 years old, landscape-type around village of domicile (ie.>50% scrub/grassland), presence of free-roaming/scavenging dogs, the number/history of dog ownership and dog carer [18]. In addition small mammal species assemblages were investigated in depth, and two dominant susceptible host species (*Microtus limnophilus* and *Cricetulus longicaudatus*) with potential for pluriannual population increases, were shown to occur in the high risk scrub/grassland habitats [4,26]. However, by 1994 the domestic dog population had crashed to almost zero throughout the region, most probably as an indirect result of rodent poisoning campaigns. The dog population only began to recover towards the end of that decade. The red fox population remained extremely small since the mid-1990s according to local farmers’ testimony. Thus, despite the identification of significant numbers of human AE cases in the 1994–97 screenings, that were mostly latent infections from 10–20 years earlier, it was considered that active peri-domestic transmission of *E*. *multilocularis* had probably ceased, and furthermore any wildlife cycle would be hardly sustainable. In 2005/2006 a third mass screening program for human AE was implemented in the Zhang/Puma area of south Gansu Province, furthermore by 2015 the dog population had fully recovered which was sampled by coprotesting. Those studies are now reported here and considered in relation to the long-term retrospective view of disease and transmission ecology.

The aim of this article is to attempt to describe the natural history and the fate of *E*. *multilocularis* transmission over >25 years in Han farmer communities of south Gansu, and to illustrate how changes in socio-environmental conditions can modify parasite transmission over the long term.

## Materials and methods

### Study site

**Fig 1:**
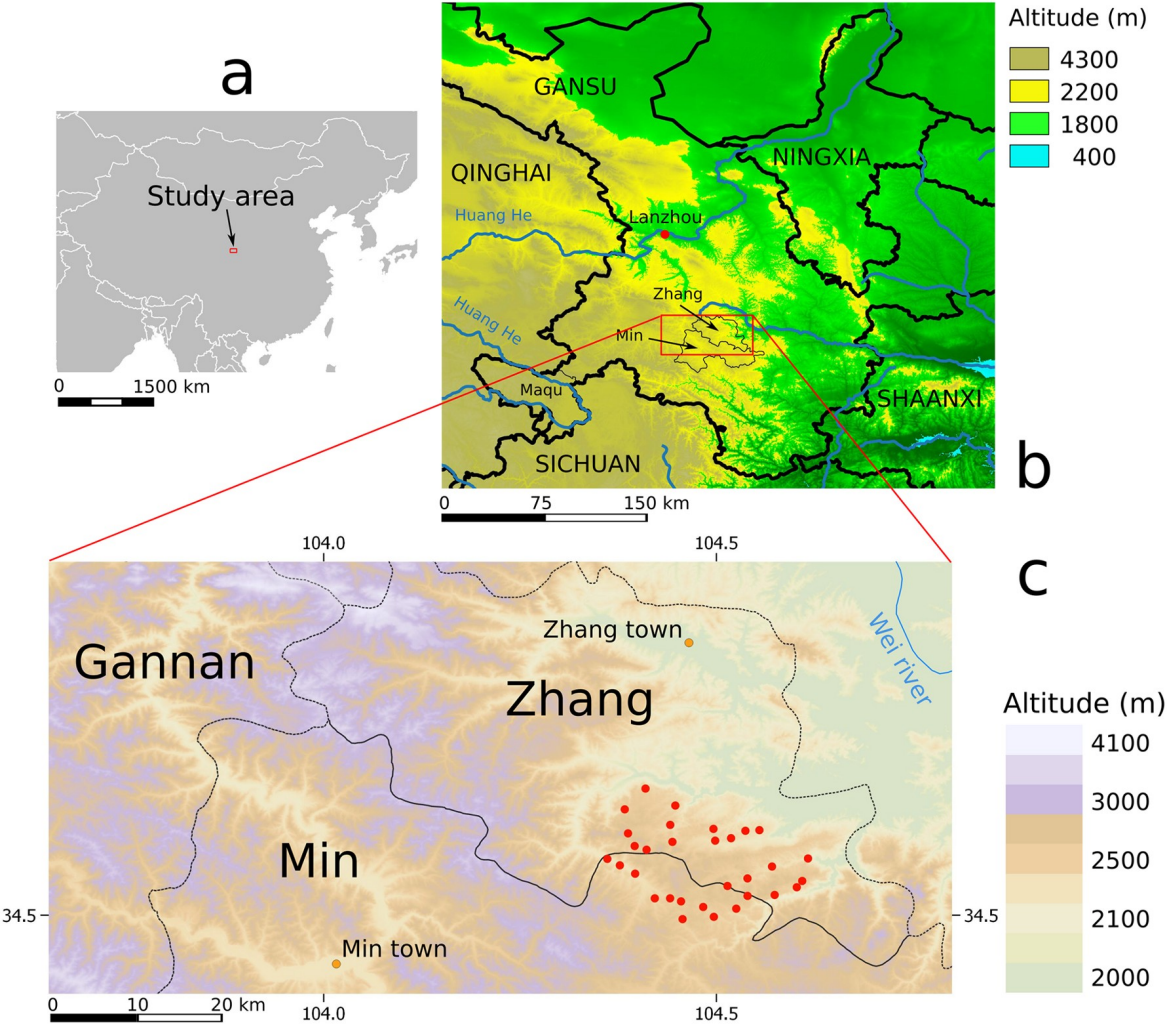
Maps of the study area. a, location in continental China; b, location among neighbouring provinces and autonomous regions; c, locations of villages (red circles): they are at some tens of kilometres from the Tibetan plateau (> 3000 m of altitude), where foci of intense transmission of *E*. *multilocularis* are reported [7,27]. Altitudes derived from ASTER Global Digital Elevation Model [28], borders from DIVA-GIS [29].

**Fig 2:**
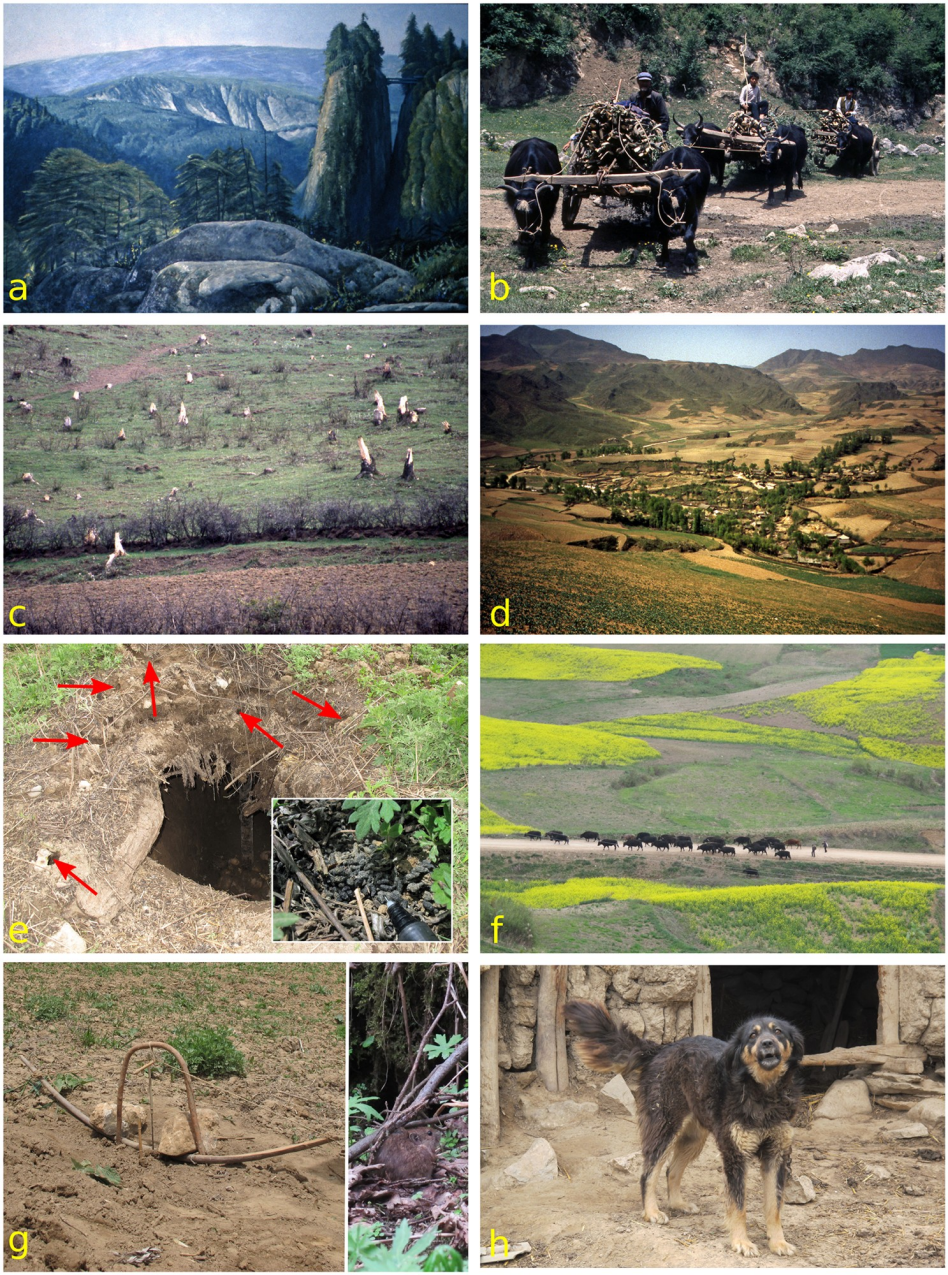
Main landscape features of the study area. a: The ancient landscape painted from GuiQing Shan (104.47°E, 34.64°N) is represented largely dominated by altitude coniferous forest (painting in a hotel in Zhang town (May 1994)); b: cattle cart carrying fire wood (July 1996); c: residual timbering (July 1996); d: landscape in May 1994: old fire tree forest was entirely timbered and mountains capped by bushes and grassland; e: potato storage in May 2015. Red arrows point to holes and galleries of *Microtus limnophilus*, easily accessible for farm dogs. Box: *Microtus* faeces. f: farmland with herd of "pian niu" (cattle-yak cross-breeds) in May 2014; g: Left, *Eospalax* sp bow trap (May 2014); right, *Ochotona* sp. in a woody area (May 2015); h: a large number of dogs were present in 2014 and 2015.

Studies were carried out in the original area of investigation that was undertaken in 1991 and 1994–97, in Zhang and Min counties of south Gansu [18] (Figs 1 and 2). The area (~350 sq. km) is characterized by 31 small discrete villages in valleys and plateau at approximately 2400–2600 m alt (kml file is provided as S1 File). The rural population comprised of greater than 98% Han Chinese, most of whom were subsistence farmers (rape, wheat, potatoes, soya) with a few medicinal herbs such as “dang gui” (当归, *Angelica sinensis*). The area is characterised by short, wet and warm summers (15–30°C) and cold winters -10 to -20°C, average yearly rainfall is 550–600 mm. Villages ranged in size from 100 to 1700 people with an average population of 350. Livestock, though not abundant, comprised of sheep and goats, pigs, chickens and small herds of cattle-yak cross breeds (“pian niu”, 犏牛).

### Human screening and AE patient follow-up

Mass screening of volunteers (self-selected) by abdominal B-ultrasound (US) scanning, complemented by serology was undertaken over the period 2005–6 (approximately 10 years after the main investigation in 1994–97). A liver scan was voluntarily performed on each person by an experienced sonographer using a portable scanner. Liver lesions/cysts if present were identified as definite, probable or query AE disease and any AE lesions classed according to the PNM classification [30]. Serological antibody testing was performed using a panel of crude native antigen extracts ie. *E*.*granulosus* cyst fluid (EgCF), protoscoleces (EgP), antigen B (EgB) and a purified specific *E*. *multilocularis* metacestode antigen (Em2) [31]. In addition, a questionnaire was administered relating to knowledge, attitudes and practices, including aspects of dog ownership.

Information about AE cases from the 1994–97 survey was followed-up in May 2014 by interviews with hospital and clinic staff in the endemic zone ie. CaoTan, Han Chuan (now Dong Chuan) and Puma, and with the head person from villages of respective patient domicile.

X^2^ was used to test the null hypothesis of categorical variable independence in contingency tables. Human AE prevalence was modelled with infection status as response variable (1/0) against independent variables using General linear model (GLM) and General additive model (GAM) with a binomial (logit) link function. Here, GAM uses cubic smoothing splines with various degrees of freedom on independent variables in order to better take possible non-linearity of the response into account [32]. Models were compared and selected using the Akaike Index Criterion [33]. Computing and graphical display were performed using R 3.5.3 [34] and the package gamlss [35].

### Dog survey

#### Collection of dog feces in the field

Dog faecal samples were collected from the ground with owner compliance from individual dogs in households, in May 2015. All faecal samples were decontaminated by deep-freezing at −80 °C for at least 7 days in order to prevent any risk of infection.

#### Questionnaires and survey methods

When collecting dog feces, a questionnaire was applied to each dog owner family, with the support of local people for translation from local dialect to Mandarin. The questionnaire included general information about the participant, such as household name, ethnicity and occupation, and dog information, such as dog age, feeding habits, whether the dogs were tied, the use of anthelmintic drug, and whether there were any stray dogs or foxes in the area.

#### DNA extraction and PCR

Total genomic DNA was extracted from feces using the QIAamp DNA Stool Mini Kit (Qiagen, Courtaboeuf, France) according to the manufacturer’s instructions, after overnight incubation of 200 mg of faeces at 56°C in 1.6 ml of lysis buffer (ASL, Qiagen).

Amplification of *E*. *granulosus* and *Taenia* spp. DNA was performed using the PCR primers Cest3 (5’–YGAYTCTTTTTAGGGGAAGGTGTG– 3’), Cest4 (5’—GTTTTTGTGTGTTACATTAATAAGGGTG—3’) and Cest5 (5’–GCGGTGTGTACMTGAGCTAAAC—3’), to target the small subunit of ribosomal RNA *rrnS* [36]. Multiplex PCR were conducted in a reaction mixture (25 μl total volume) consisting of 3 μl of DNA extract (10–100 ng/μL), 1 × HotStarTaq Master Mix (Qiagen), 0.2 μM of Cest3 and Cest4, 0.4μM of Cest5, and PCR-grade water. A negative control (PCR-grade water) was introduced every 10 samples. The amplification cycling program consisted of a 15 min activation step at 95°C, followed by 40 cycles of denaturation at 94°C for 30 s, annealing at 58°C for 1 min 30 and extension at 72°C for 1 min, with a final extension at 72°C for 10 min. The PCR products (expected size of 117 bp for *E*. *granulosus* and 267 bp for *Taenia* spp.) were separated and visualized using the QIAxcel device (an automated capillary electrophoresis system produced by Qiagen) using a QIAxcel DNA high-resolution kit (Qiagen). PCR-positive products were purified using a QIAquick PCR purification kit (Qiagen) according to the manufacturer’s instructions. Direct sequencing of the PCR products was performed as recommended by Trachsel et al. (2007; *i*.*e*., using the primers employed for the PCRs, excepted for *Taenia* spp. which were sequenced using Cest5_seq_), with an automated sequencer (Applied Biosystems 3130 Genetic Analyzer). A homology search of the sequences generated in this study was then performed by conducting an online search with the NCBI Basic Local Alignment Search Tool for nucleotides (BLASTN) in the GenBank database (http://blast.ncbi.nlm.nih.gov/Blast.cgi).

To detect and quantify the presence of *E*. *multilocularis* DNA in the tested samples, a real time quantitative PCR (qPCR) was performed as previously developed [37], by amplifying a part of the large ribosomal subunit gene (*rrnL*). To identify the presence of PCR inhibitors, the internal control Alea was added to the PCR mixture, by performing a duplex PCR (Em/Alea qPCR) [38]. The duplex qPCR was performed in a 20 μl reaction mixture containing 10 μl of 2 X TaqMan Gene Expression master mix (Life Technologies, Foster City, CA), 5 pmol of *E*. *multilocularis* primers (rrn-Em-fwd 5’—CTGTGATCTTGGTGTAGTAGTTGAGATTT—3’, rrn-Em-rev: 5’—GGCTTACGCCGGTCTTAACTC—3’), Alea primers (Alea-fwd: 5’—CCTAAAAATGTCTATGATTGGTCCACTA—3’, Alea-rev: 5’—GGGAGTACCTTGCCATACAAAATT—3’), 0.4 pmol of the hydrolysis *E*. *multilocularis* probe (5’—FAM-TGGTCTGTTCGACCTTTTTAGCCTCCAT-TAMRA) and Alea probe (5’—VIC-TTAAATCAACTCCTAAATCCGCGCGATAGG-TAMRA—3’), and 5 μl of total extracted copro-DNA added to this mixture. The qPCR was run on a model 7500 Fast real-time PCR system (Life Technologies, Foster City, CA) for 45 cycles. All PCRs were performed in duplicate, and results were expressed as quantitative cycle (Cq) numbers.

### Ethics statement

1994–96 and 2005–6 human screening programs were ethically approved by the relevant authorities of the Lanzhou Medical University. From 1994–96 AE cases were offered a free 6 months course of albendazole (ABZ), referral to the local medical centre or hospital and subsidized surgery for relevant cases [18]. From 2006/2007, the Ministry of Health provided both free ABZ-based treatment and heavily subsidized surgical intervention if deemed appropriate. All adult subjects provided written informed consent, and a parent or guardian of any child participant provided informed consent on the child’s behalf.

All animals were handled in strict accordance with good animal practice according to the Animal Ethics Procedures and Guidelines of the People’s Republic of China, and from 2012 the study was approved by the Animal Ethics Committee of Lanzhou Veterinary Research Institute, Chinese Academy of Agricultural Sciences (No. LVRIAEC2012-007).

## Results

### AE screening and patient follow-up

#### 2005–6 screening

In total 2500 people were included in the 2005–6 ultrasound mass screening survey. One AE case of 74 years old was identified from the 1991 survey. In addition, 11 people were also historic AE cases, detected in 1994–6. Among them, two were cured after surgery and one case spontaneously or possibly due to ABZ therapy. These three cured AE cases were not included in the 2005–6 cohort. In total, 28 AE cases were diagnosed with a mean case age of 40 years. Among these an 8 year old boy (born in 1997 or 1998) was diagnosed with an early hepatic AE lesion staging P2 (2 x 1.5 cm^2^) according to the PNM WHO clinical classification [30] which suggests probable infection in the late 1990s or early 2000s. One other AE case was born in 1992 or 1993, during a period when the dog population was diminishing. The other 26 AE cases were born before 1992, hence had an age compatible with infection during the period when a large dog population and dog infection with *E*. *multilocularis* was evidenced [25]. However, more recent human infection cannot be formally excluded.

Eleven persons (0.4%) declared they had contact with foxes, including 10 under 19 years old. All were born before 1994, but considering the fox population was very low (almost extinct) in the early and mid-1990s [4], this might indicate that contacts declared by young persons probably occurred from the late 1990s or the early 2000s onwards when the fox population was recovering. Furthermore, 423 people (17%) declared to own a dog, indicating also a recovery of the dog population. The 8-year-old AE case mentioned above indicates that the parasite was present in the recovering fox or dog population in the late 1990s or early 2000s.

#### Comparison of AE case data between the 2005–6 and 1994–6 screenings

**Fig 3:**
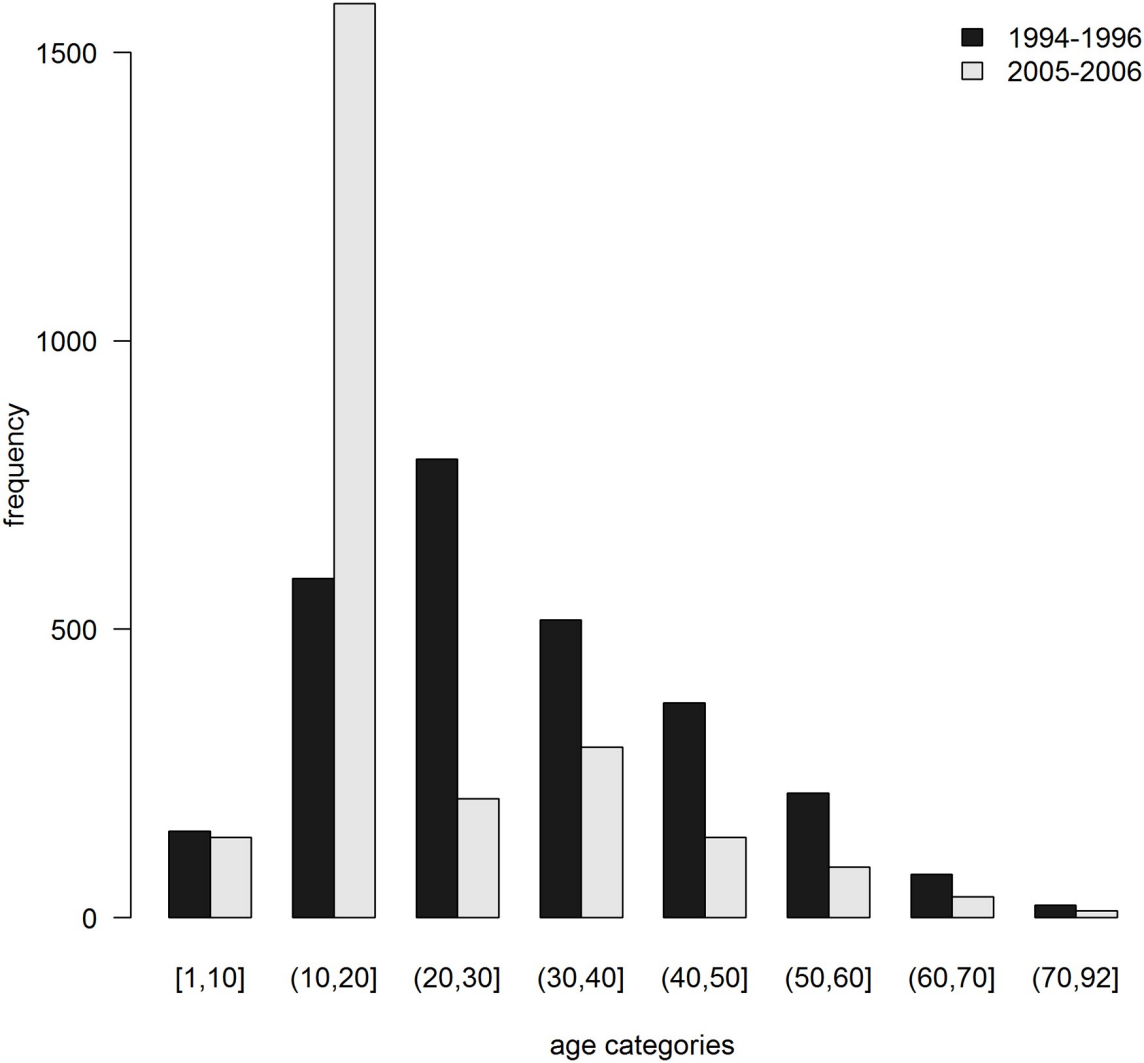
Number of people examined according to age categories.

**Fig 4:**
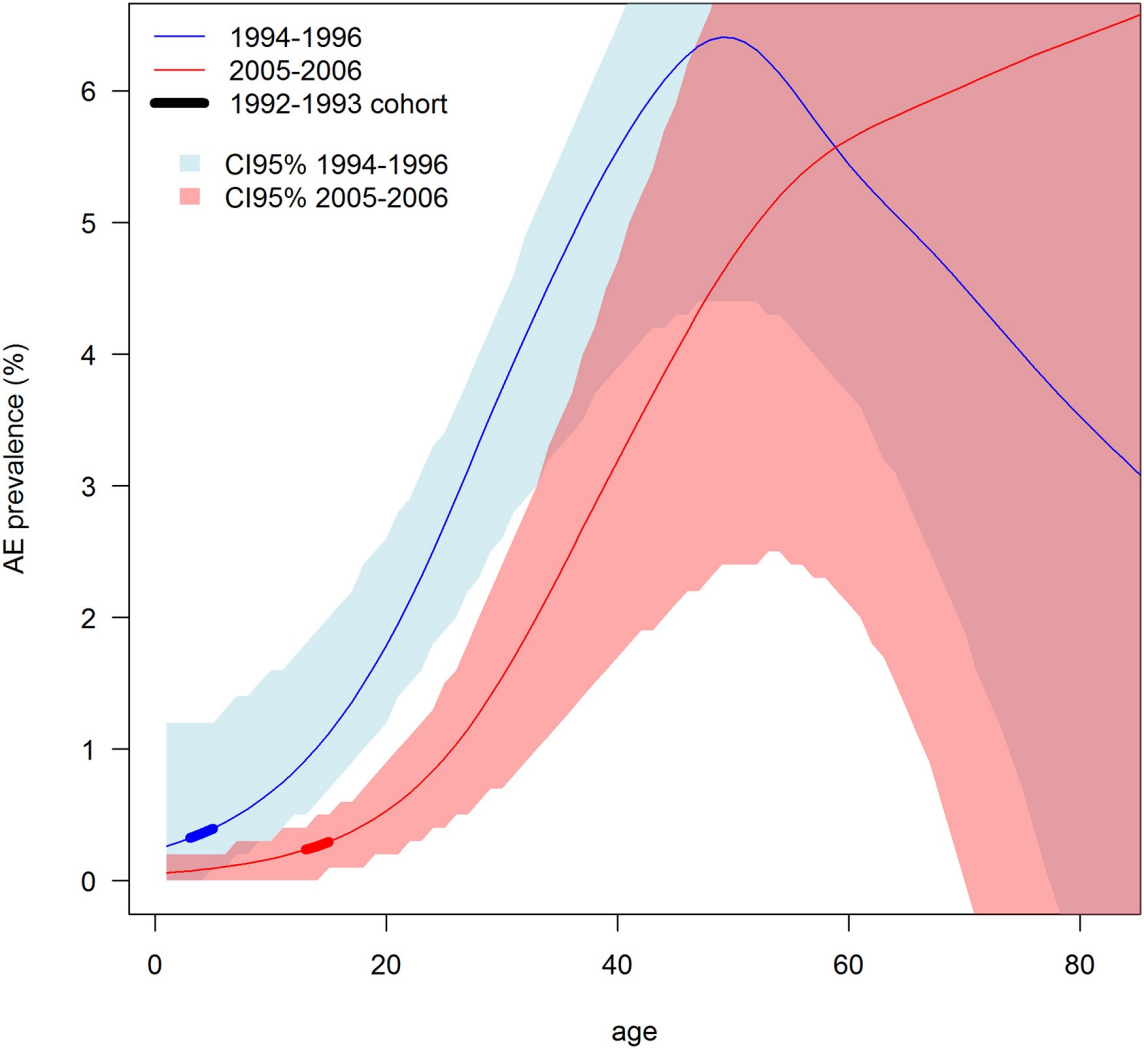
Age specific prevalence in populations screened in 1994–1996 and 2005–6. The thick line materialise the fate of the 1992–1993 cohort born just after 1991 (year when dogs were still infected [25]) and before 1994 (year when the dog population was virtually extinct [18]).

Overall prevalence of human AE in the 2005–6 and 1994–6 mass screenings could not be compared globally due to different sample sizes in each age category (Fig 3). As a consequence, infection status observed in the 2005–6 and 1994–6 screenings were modelled against age in each case for further comparisons. GAM showed a lower AIC than GLM in both cases and were therefore selected (see S2 File), supporting the notion of AE prevalence varied non-linearly with age (Fig 4). Moreover, age-specific AE prevalence for the 6 to 38 year old cohort was significantly larger in the 1994–6 sample than in 2005–6 (Fig 4 and Table 1), with an average odds ratio of 3.6 (CI95% 1.7–17.2) during this time span. We failed to detect differences between the two screenings in the older age categories.

**Table 1:** Age specific odd ratios based on the two models of Fig 4.

age	odd ratio	CI95%
10	5.59	1.7–29.9
20	3.66	1.9–8.2
30	2.29	1.4–4.3
40	1.63	1–2.9
50	1.30	0.8–2.4
60	1.09	0.5–2.5
70	0.96	0.3–4.7

#### 2014 AE case follow-up

**Fig 5:**
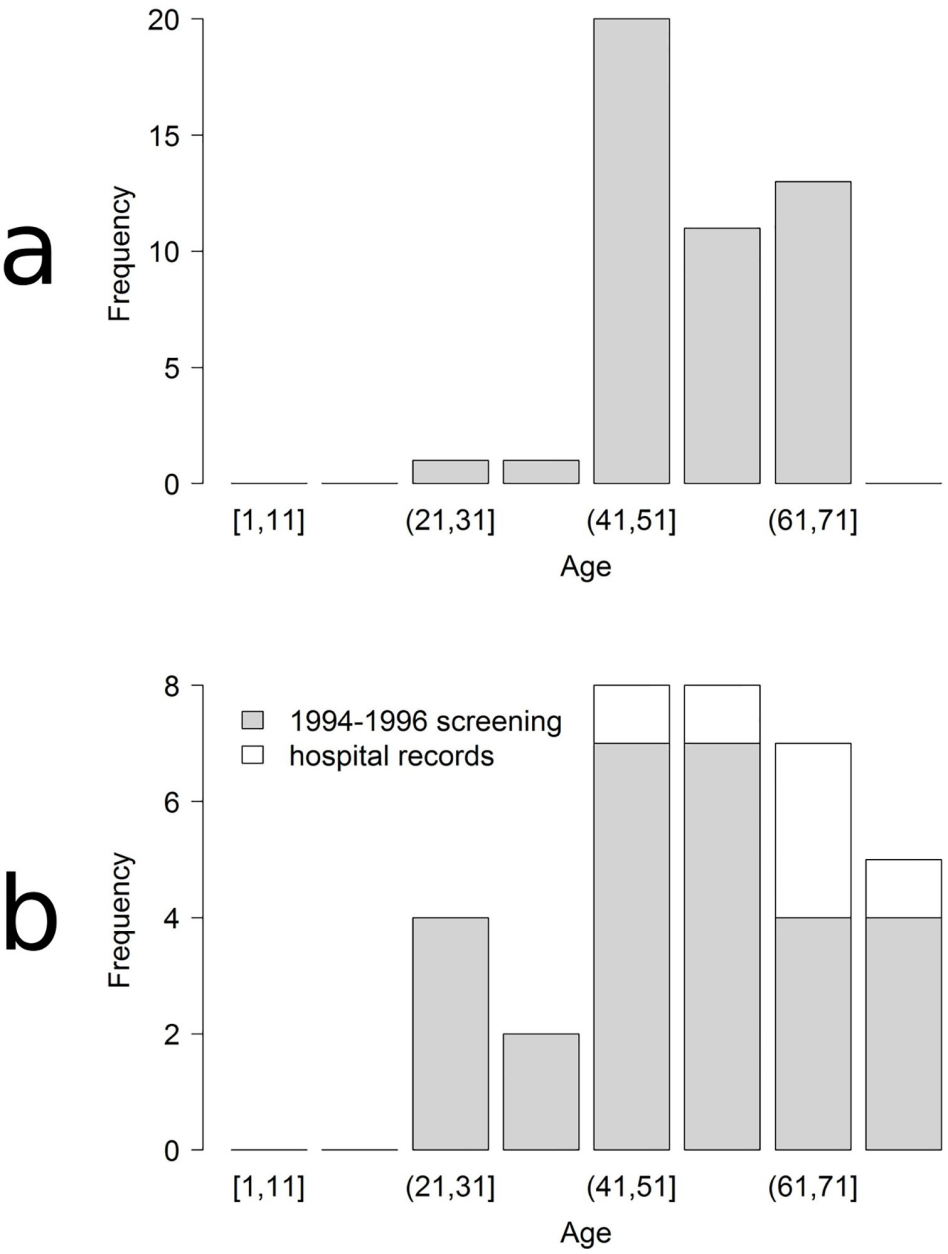
Age structure of patient population. a, age at death of patients died before May 2014; b, age of patients still alive in May 2014.

In May 2014, a total of 97 confirmed AE cases could be identified in the follow-up. This included 14 AE patients in Cao Tan and Puma hospital records whom appeared not to have been screened in 1994–6. In addition, among those diagnosed in the 1994–6 screening (n = 77), 59.7% were still alive in May 2014, indicating a surprising longevity for patients with this potentially fatal parasitic disease. Fig 5 shows the age structure of the population still alive and the age at death of the AE patients who died before May 2014. The two youngest AE patients alive in 2014 were both male first identified in 1996 when they were 12 and 18 years old respectively. At that time, the former had a 5 cm hepatic lesion judged abortive and the latter a lesion of 3 cm. Both were strongly seropositive in 1996. Hepatic lesions in other AE patients ranged from abortive (n = 6) to 15 cm diameter (median 3 cm) when detected in 1994–6. In 2014, in hospital records, we did not find AE cases younger than 35 years old at diagnosis (range 35–76 years) among those patients who were not included in the 1994–6 screening. Reliable data on 28 AE cases that died before 2014 indicated that the average number of years of case survival after initial diagnosis was 8.7 years (range 1–18 years).

### Dog survey 2015

#### Questionnaire

**Fig 6:**
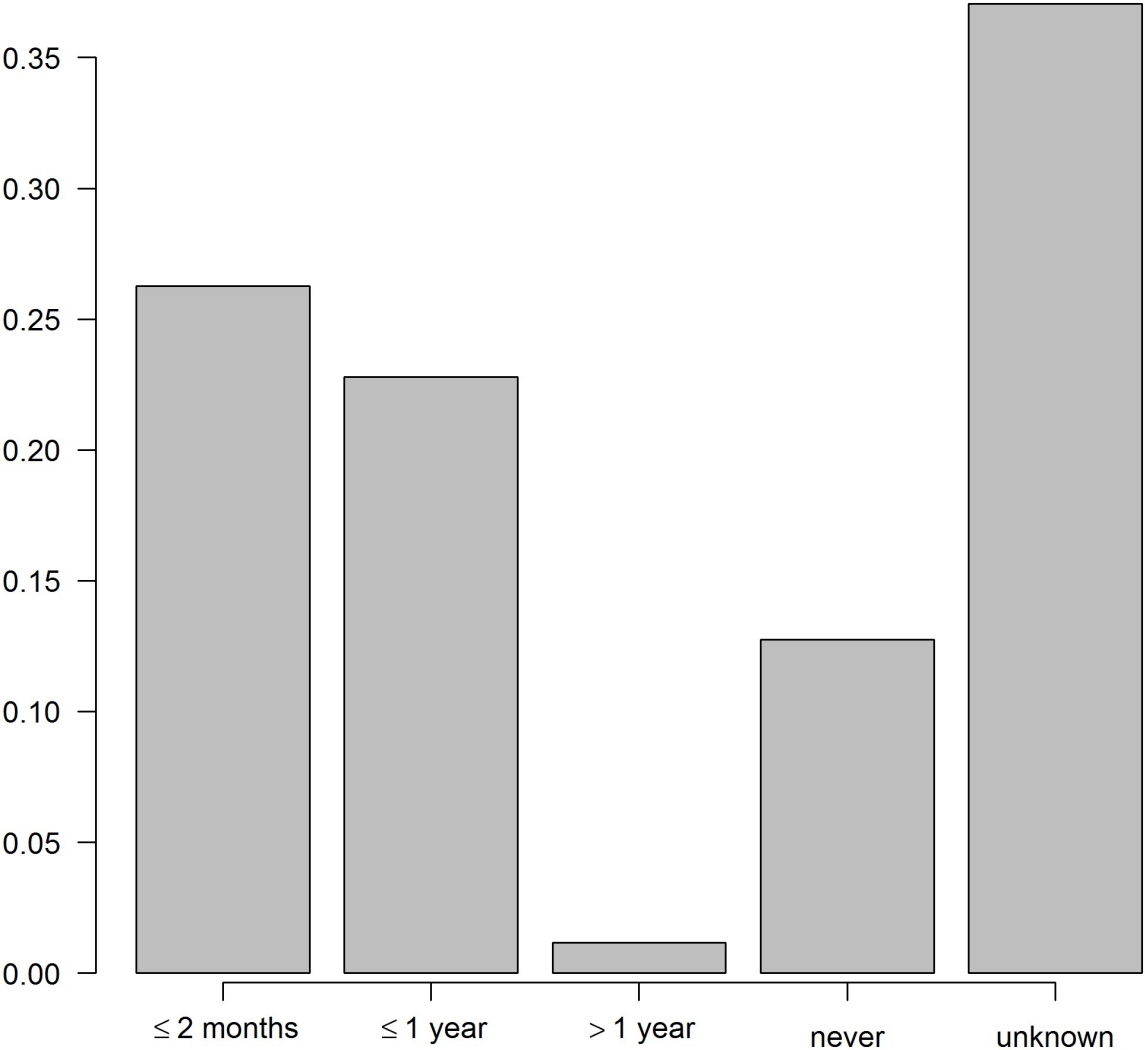
Frequency of praziquantel treatments in dogs (2015 survey).

In May 2015, 257 questionnaires were applied in 26 villages of those screened in the 1990s. People interviewed were Han farmers. They owned 1–2 dogs (mean = 1.3, standard deviation = 0.45). 66% of the dogs were declared to be always tied and 8% never. 62% of dogs were obtained within the community and only 16% were bought in markets. Dog geographical origin was most often unclear, and possibly given by neighbors or villages nearby, but in two cases dogs were declared to be purchased more than 200 km distant, in Maqu county Gannan Tibetan Autonomous prefecture (Fig 1). Fig 6 shows that anthelminthic praziquantel (PZQ) distribution although not very systematic, was applied with near 50% dogs`last treatment being less than 1 year previously, and more than 25% of dogs dosed less than two months prior. The local government CDC gave the PZQ tablets in 96% of cases reported. Furthermore, 24% of the people interviewed declared to have seen a fox recently.

#### DNA screening of dog faecal samples

In total 256 dog feces were collected and tested using DNA targets. We did not detect DNA of *E*. *multilocularis* or *E*. *granulosus* (Table 2). The prevalence of the non-zoonotic cestode *Taenia hydatigena* by copro-DNA testing in 2015 was more than 100 times smaller than by necropsy in 1991 (0.4%, CI95% 2–2.5%, *versus* 43.1%, CI95% 30.4–56.7%).

**Table 2:** CoproDNA based prevalence of cestodes in owned dogs (n = 256) from Zhang and Puma counties (May 2015).

Species	Number positive	Prevalence	Confidence interval 95%
*E*. *multilocularis*	0	0	0–1.8
*E*. *granulosus*	0	0	0–1.9
*Mesocestoïdes* sp.	2	0.78	0.1–3.1
*Taenia hydatigena*	1	0.39	0.02–2.5

## Discussion

Human alveolar echinococcosis caused by infection with *Echinococcus multilocularis* is one of the most potentially pathogenic helminthic zoonoses. Transmission occurs only in the northern hemisphere primarily involving wildlife cycles typically between fox and small mammal intermediate hosts. In the late 1980s/early 1990s we identified a large focus of human AE in resource-poor upland agricultural communities in south Gansu Province, China [25]. More detailed investigations in 1994–97 expanded community screening and identified key risk factors of dog ownership and landscape type around villages that could support susceptible rodent populations [26,4,18]. We subsequently undertook follow-up eco-epidemiological studies in 2005/6 and in 2014.

The 2005–2006 community mass ultrasound screening in Zhang and Puma areas showed a decrease in AE prevalence in the human population, which was most marked in younger age categories indicating that they were less exposed to AE infection. Some human AE infection post-1995, however was evidenced after community mass screening by at least one case < 10 years of age, indicating that the parasite *E*. *multilocularis* was still circulating in the late 1990s or early 2000s. In May 2014, we could not identify in hospital records any “new” AE patients (not included in the 1994–97 screening) that were born after 1997. The youngest of the AE cases was 35 years old at diagnosis, hence had an age compatible with an infection prior to 1994.

**Fig 7:**
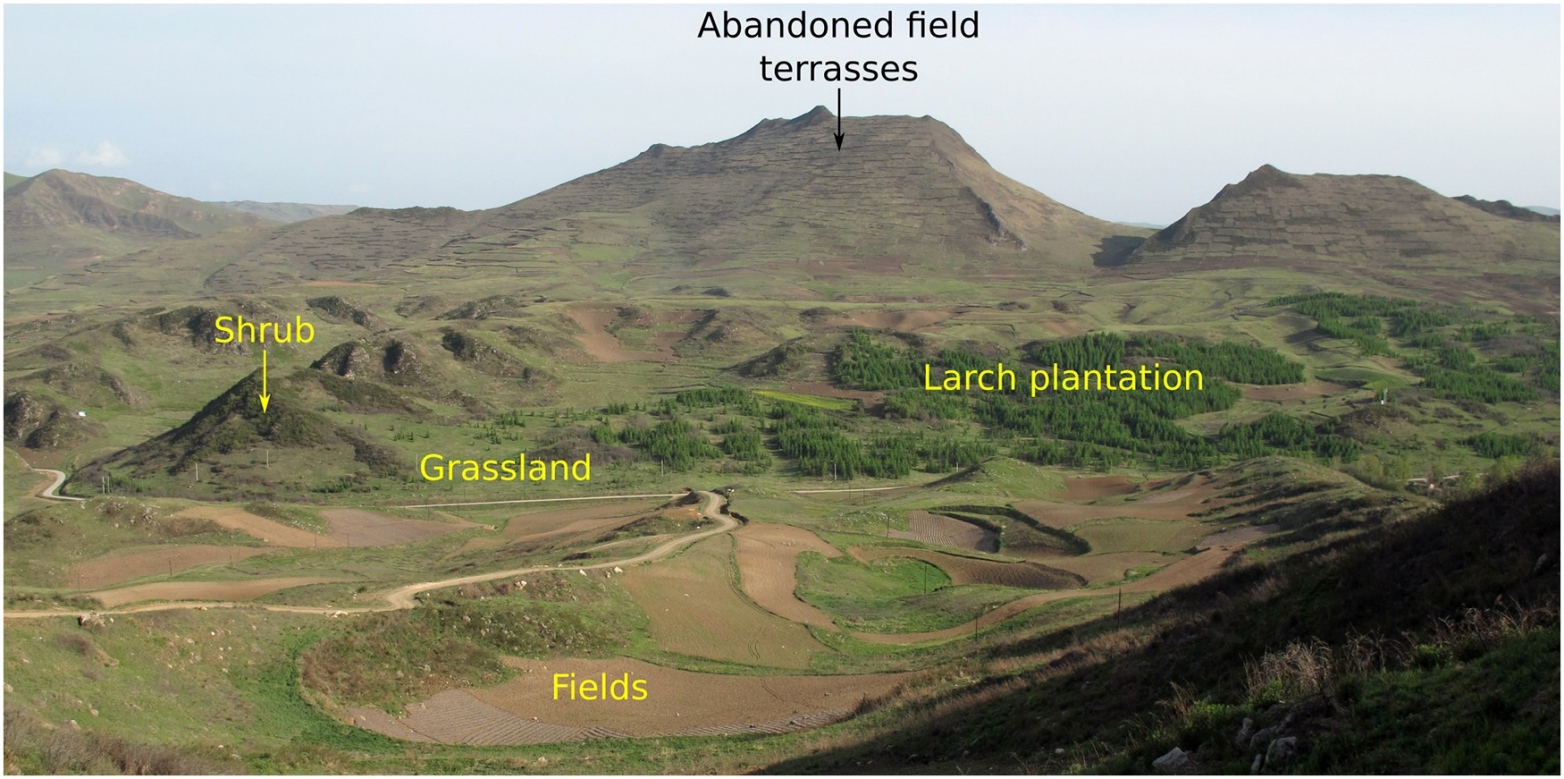
Landscape in May 2015 on the way from CaoTan to SanYanQuan. Ploughing has been banned on former field terraces on steep slopes now left to natural vegetation recolonization, and farming is restricted to flat areas. Reforestation programmes have additionally been carried out since the mid-1990s.

In May 2015, in contrast to the period 1994–97, a large number of owned dogs were present in villages in the endemic area with most of them tied, a fraction treated with PZQ, but some were allowed to free-roam and possibly eat small mammals. A large number of small mammal indices (species of the genera *Microtus*, *Cricetulus* and *Eospalax*) could be observed during the 2015 survey, at similar frequencies to those observed in the 1990s (Giraudoux P., personal observations, see Fig 2e and 2g). This supports the notion that a peri-domestic dog–small mammal life-cycle for *E*. *multilocularis* could still be potentially completed in the area, at least by a proportion of the dog population. Moreover, the fact that some dogs had been imported from Maqu county (Gannan Tibetan Autonomous Prefecture) increased the risk of possible re-introduction of *E*. *multilocularis* infection from distant endemic areas of the eastern Tibetan plateau [27]. Furthermore, since the late 1990s, soil protection and reforestation programmes have largely extended bush and forest areas on slopes too steep to be cultivated without deleterious erosion (Fig 7). This provided more potential habitats for wildlife including red foxes (*V*. *vulpes*), the latter being more frequently observed by farmers in 2015 than in the 1990s, when the red fox was believed to be virtually extinct in the area [4]. Nevertheless, we did not find any evidence for *E*. *multilocularis* infection in dogs, indicating that a peri-domestic cycle had likely not re-emerged by 2015. The slow progressive recovery of the dog population post 1997, with a probable low or zero infection rate, combined with a small fox population, might have locally interrupted the parasite life-cycle, and kept transmission very low for a period after the early-1990s. This could explain the decrease in human AE prevalence observed in 2005–2006 in younger age categories. In 2005, a National Echinococcosis Control programme was initiated in western China, and included improved surveillance (and treatment access) of human disease and regular deworming of dogs [39].

It is now well recognized that anthropogenic land-use changes drive a range of infectious disease outbreaks and emergence events and modify the transmission of endemic infections. These drivers include agricultural encroachment and deforestation [40]. Patz et al. [41] have proposed a systems model echoing those proposed by WHO [42] and MEA [43]. It includes specific health risk factors, landscape or habitat change, and institutional (economic and behavioural) levels and their health consequences. Various levels of investigation and intervention were described and ranged from specific risk factors and determinants of population vulnerability, to larger institutional and economic activities. Such conceptual models have been specifically considered for *E*. *multilocularis* transmission and human AE [4,44,8,10]. They confirm the inextricable linkages between environmental, socio-economic and proximal behavioural factors influencing the transmission dynamics of this helminthic zoonosis. All those models lead to the notion that, not one main factor alone, but a combination of factors, convergent or concurrent, affect the transmission dynamics of *E*. *multilocularis* in a nested hierarchy of time and space [44]. In such complex transmission systems, there is little hope for isolating a single aspect that would explain transmission ecology for example in the Zhang/Puma area of western China. However, accepting a form of holistic approach from the start, one can isolate subsystem groupings of predominant factors that altogether help to explain transmission patterns in an area for a given time span and scale, and to identify the tipping points when one shifts from one subsystem to the other.

Combining climatic, land cover and intermediate host species distribution data, Giraudoux et al. [6] have identified and mapped four spatially distinct types of regional transmission ecosystems for *E*. *multilocularis* in China, typified by the presence of one of the following small mammal ‘flagship’ species: *Ellobius tancrei*, *Ochotona curzoniae*, *Lasiopodomys brandtii* or *Eospalax fontanierii*. The Zhang/Puma study area typically belongs to the latter. Furthermore, using data from a community mass-screening screening on the Tibetan plateau, Giraudoux et al. [7] applied general additive linear models and found that human AE was spatially correlated with landscape features and climate which could confirm and predict human AE disease hotspots over a 200,000km^2^ region of the Tibetan plateau. Notably, four areas of human AE risk that were not in the data set used for training the model were predicted, including the Zhang/Puma AE disease hotspot [7]. This indicated that driving forces relying on climate, landscape and small mammal communities assessed on a large scale, combine to make transmission possible on the local scale (~400 km^2^). On this scale, in Zhang/Puma, four small mammal assemblages were identified in specific habitats of a deforestation gradient i.e. forest, shrubland and grassland, farmland and village [26]. All of the 10 species forming those small mammal assemblages were potential intermediate hosts for *E*. *multilocularis*. However, a significant association between human AE prevalence and land area under shrubland or grassland was found, indicating that, on that scale (~100 km^2^), the average density of small mammal intermediate hosts was likely a key factor explaining *E*. *multilocularis* transmission intensity [4]. The differences between small mammal habitats may be explained by the fact that mature forests, although richer in species biodiversity, cannot sustain high densities of small mammals on a large scale, compared to grassland and shrubby habitats. In these latter habitats, some rodent species prone to cyclicity can thrive at very large population densities (e.g. [45,46,44]), and importantly with comparatively low vegetation, they are more easily accessible to fox and dog predators [47]. Farmland, due to the high productivity of agrosystems can also provide temporary favourable habitats for high densities of small mammals, but tilling and seasonality of resources are often strong limiting factors. Furthermore, transmission of *E*. *multilocularis* depends also on the density of definitive hosts [48] and on human behaviour [18]. For instance, differences in living standards can explain why with similar prevalence of *E*. *multilocularis* in fox populations, human AE prevalence is more than 40 times lower in high endemicity areas of central Europe [49] compared to the eastern border of the Tibetan plateau [27]. Any change in one of those factors or set of factors can lead to change in transmission intensity.

During this retrospective >25 year overview in the south Gansu endemic zone, a number of economic and environmental changes were observed in the study area. Using Landsat Multispectral Scanner (MSS) and Thematic Mapper TM data, Danson et al. [50] confirmed that there had been an expansion of agricultural land from approximately 41% in 1975 to 58% in 1997. There was also an apparent reduction in secondary forest from 21% to 14%. In addition, there was generally a strong negative correlation between the area of secondary forest and the area of agricultural fields. There was also a strong positive correlation between the area of forest and the area of tree/shrub, showing the close relationship between forest and shrub clearance that is necessary to establish agricultural expansion. This was consistent with reports from local people in the Zhang/Puma area, and in particular the expansion of ploughed fields at the expense of semi-natural vegetation and wildlife. Bears (*Ursus thibetanus*) were still present in this region of south Gansu in the 1980s as testified by skins found in houses in the early 1990s. One of us (Craig, personal observation) observed one wild takin (*Budorcas taxicolor*) caught by people close to Han Chuan village (Zhang county) in 1991, and in 1994 local people were reporting that leopard (*Panthera pardus*) was still found some ten years before in the GuiQing Shan valley close to Cao Tan also in our study area. All those large mammal species were extinct by 1994 when landscape was then characterised by large areas of ploughed fields including on steep slopes (> 30°), by hills capped by bushes and grassland, and by the last timbering sites (Fig 2). However, considering the environmental issues raised by agricultural encroachement everywhere in China, the Chinese government enforced a forest and soil protection policy from the mid-1990s onwards. As a result strict controls led to a ban of tilling on steep slopes, and the setting aside of large areas that were abandoned and then colonized over some years by a succession of natural vegetation from grassland to shrub, or deliberately replanted mostly with larch (*Larix sp*.) (Fig 7). Another key event in the Zhang/Puma area, as already mentioned, was a dog and fox population crash in 1992–1993. We did not find a clear explanation for this event, the most likely being a side effect of agricultural expansion when massive rodent poisoning campaigns were carried out for crop protection, with deleterious non-intentional impacts on dogs and wildlife.

**Fig 8:**
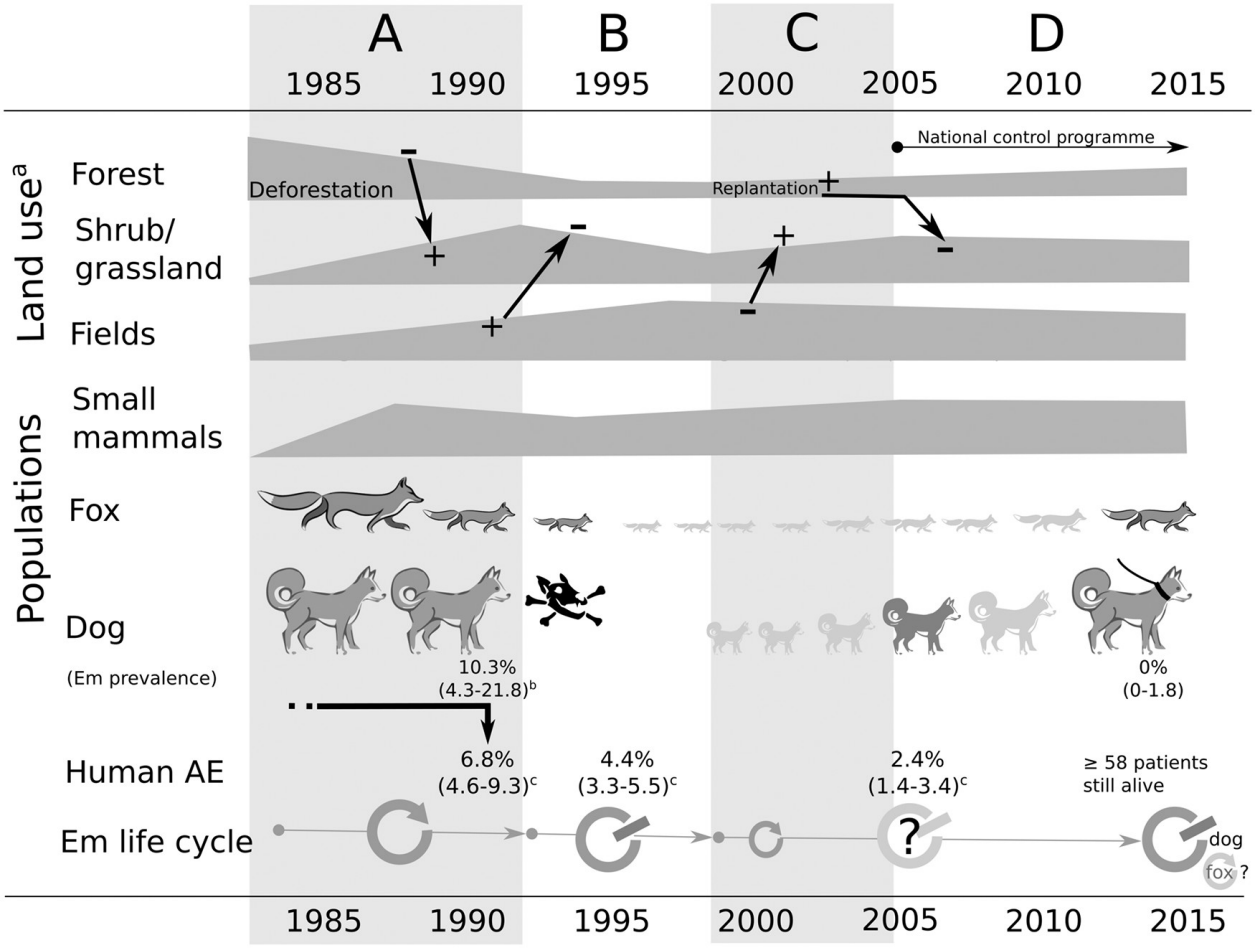
Change scenario in the transmission system from the 1980s to 2015. a, after [50,4]; b, after [25]; c, based on [25] and the present study (AE prevalences are adjusted at 35 year old). A. **Agriculture encroachment and deforestation (1970s-early 1990s)**: this period corresponds to an increase of forest exploitation with subsequent increase of pastureland, tilled areas, and associated human and dog populations (71% agricultural families kept dogs in 1991 [25]). Zokors (*Eospalax fontanieri*) were trapped by farmers to protect crops, and some were fed to dogs. During this period, the red fox was still relatively abundant and an increase of shrubland and grassland occurred, and these habitats were favourable to small mammal species prone to reach high densities (e.g. *Microtus limnophilus*, *Cricetelus longicaudatus*). These susceptible rodent host species were easily accessible to dogs compared to forest dwelling small mammal species, and thus created conditions extremely favourable to intensive transmission. This led to ~10% prevalence of *E*. *multilocularis* in dogs and a human AE prevalence of ~7% by 1991. B. **Dog and fox population crash (1992–97)**: the dog and fox population crash (15.2% of people screened declared to have owned a dog before the crash) occurred in 1992–1993 and the virtual extinction of the population was observed from 1994 to 1997. It most likely significantly interrupted the *E*. *multilocularis* lifecycle. Human AE cases identified in 1994–1996 have probably all been infected in `period A`and an average of ~4% human AE prevalence is still observed. Furthermore, human AE prevalence was three times lower in areas where villages were surrounded by a smaller ratio of shrubland and grassland [4]. Large and medium-size mammals, including foxes, are virtually extinct by 1994. C. **Timbering ban and forest replantation (1998–2006)**: forest regulations enforce a tilling ban on steep slopes and tree replantation occurs. This leads to a decrease of farmland and an increase of areas recolonized by natural vegetation (grass, bushes), and eventually to result in an increase in the area of forest patches. The fox population (a number of young people declared they had contact with foxes in 2005–2006) and dogs (17% people screened declared to have dog in 2005–2006) gradually recovers. At least one young AE case infected during this period was identified indicating that *E*. *multilocularis* transmission had re-emerged either in the fox or in the dog population or both. However the shift to lower human AE prevalence in the younger age categories of the 2005–6 screening (Fig 4) indicated that zoonotic transmission to humans was much lower than in the past. D. **National Echinococcosis Control programme (2007–2014)**: most dogs were leashed and a large fraction of the dog population was treated with the anthelminthic praziquantel, but not as frequently as recommended. No evidence of *E*. *multilocularis* infection was found in the 2015 dog survey, and a very low prevalence (~0.4%) of *Taenia hydatigena* was found, contrasting with the 43% found in 1991 [25]. This indicates either the efficiency of PZQ treatment also towards this parasite, and/or that dogs have reduced access to raw offal from livestock (e.g., sheep, goats, and pigs). However, a trade in guard dogs over this period increased the potential risk of reinfection from other endemic areas (e.g. the Tibetan plateau). 59.7% of human AE cases identified in 1994–6 were still alive in 2014, and little evidence of recent infection was indicated in the human population.

Considering the complexity of such transmission systems, human AE disease risk and the possible variations of the driving forces over years, it is difficult to assess retrospectively the sequence of events that drove changes in transmission intensity of *E*. *multilocularis* since the 1980s. This is further complicated by the generally slow (several years) but varying pathologic development of the parasite in humans, which generally prevents precise estimation of the time of human infection. However, we consider that even if major gaps exist in the decadal data sets for the Zhang/Puma area, we can focus on four probable key periods and tipping points (A-D in Fig 8) described below.

In summary, the current study is one of the few to attempt to explain the long-term transmission ecology and epidemiology of human alveolar echinococcosis in a highly endemic resource-poor region of Eurasia and in western China in particular. Early investigations in the 1990s, in the Zhang/Puma area of south Gansu, identified the dog as the key zoonotic risk and an important peri-domestic definitive host for *E*. *multilocularis*. In addition a key role was highlighted in this region for the existence of `risky`or `unhealthy`landscapes [41], characterised by grass and low shrub which was largely created by rapid deforestation and agricultural expansion from the 1970s, which subsequently enabled significant population growth of 2 or 3 susceptible intermediate host rodent species. Due to the long asymptomatic period of human AE the large numbers of cases detected between 1994–96 were probably infected 10–20 years before when high-risk landscapes, large dog populations and an increase in human settlements became a critical tipping point in the local transmission ecology. As further intensification of agriculture progressed in the 1990s the area of ploughed land increased and concurrently areas of grass/shrub habitats decreased. At this time, farmers also started application of newly available rodenticides, which killed rodent pests but also accidentally dogs by secondary poisoning. Together these factors resulted in a reduction in peri-domestic transmission of *E*. *multilocularis* and reduced zoonotic risk for many communities so that by 2006 almost all AE cases were older than 30 years. By 2015, although the dog population had recovered the parasite had not re-emerged in dogs, and together with a government dog dosing programme, peri-domestic transmission of *E*. *multilocularis* had virtually ceased. Considering the high biotic potential of *E*. *multilocularis* [51], this current low transmission situation may however be threatened (i) by reforestation programmes that may lead to appearance of temporary risky landscapes, (ii) by the translocation of dogs from the highly endemic region of the eastern Tibetan plateau, and (iii) by the cessation of PZQ treatments and control policy of dogs in villages [52].

Finally, although we observed that the mean survival period for those AE cases that had died before 2014 was only 8 years, we found that approximately 60% of AE cases were still alive in 2014 after 20 years, most probably in large part due to the application of successful community-based long-term ABZ therapy for AE cases.

## Supporting information

S1 File Location of the villages of the study area (kml file). (ZIP)

S2 File Model selection details. (DOCX)

S3 File STROBE Checklist. (DOC)
