# Supplementary material for: Long-term retrospective assessment of a transmission hotspot for human alveolar echinococcosis in mid-west China
Source: PLoS Negl Trop Dis. 2019 Aug 30;13(8):e0007701. doi: 10.1371/journal.pntd.0007701 (PMC6742415; doi:10.1371/journal.pntd.0007701)
Supplement: S2 File — (DOCX) [file pntd.0007701.s002.docx]

Supplementary Material: model selection

PG

25 mai 2019 17:28

library(gamlss, quietly=TRUE)
library(pgirmess)
load("ModSel.Rdata")

The response variable is non-infected/infected, 0/1 (hence binomial), but might not be linear. Two types of model are be compared, GLM (assumes the linear predictor to be linear) and GAM (relaxes the assumption of linearity on the overall range of values and uses a df parameter to smooth the model to data to local linearity (= model non linearity) with, basically, a series of linear model with a “smoothing” parameter using a cubic smoothing splines function). In the GAM case, fitting depends on a degree of freedom with a risk of overfitting if the df number is too large. Akaike Index Criterion is used to select an “optimal model”.

# 2005-2006 screening

modz1<-gamlss(aePG~age,family=BI,data=zhao[,c("age","aePG")])

## GAMLSS-RS iteration 1: Global Deviance = 273.7466
## GAMLSS-RS iteration 2: Global Deviance = 273.7466

modz2<-gamlss(aePG~cs(age,df=1),family=BI,data=zhao[,c("age","aePG")])

## GAMLSS-RS iteration 1: Global Deviance = 264.5581
## GAMLSS-RS iteration 2: Global Deviance = 264.5581

modz3<-gamlss(aePG~cs(age,df=2),family=BI,data=zhao[,c("age","aePG")])

## GAMLSS-RS iteration 1: Global Deviance = 262.5928
## GAMLSS-RS iteration 2: Global Deviance = 262.5928

modz4<-gamlss(aePG~cs(age,df=3),family=BI,data=zhao[,c("age","aePG")])

## GAMLSS-RS iteration 1: Global Deviance = 261.1645
## GAMLSS-RS iteration 2: Global Deviance = 261.1646

AIC(modz1,modz2,modz3,modz4)

## df AIC
## modz2 3.000299 270.5587
## modz3 4.000530 270.5939
## modz4 4.999347 271.1633
## modz1 2.000000 277.7466

selMod(list(modz1,modz2,modz3,modz4))

## model LL K N2K AIC deltAIC w_i
## 2 cs(age, df = 1) -132.2790 3.000299 833.2503 270.5587 0.00000000 0.36
## 3 cs(age, df = 2) -131.2964 4.000530 624.9172 270.5939 0.03519582 0.36
## 4 cs(age, df = 3) -130.5823 4.999347 500.0653 271.1633 0.60466060 0.27
## 1 age -136.8733 2.000000 1250.0000 277.7466 7.18790469 0.01
## AICc deltAICc w_ic
## 2 270.5683 0.00000000 0.36
## 3 270.6099 0.04161465 0.36
## 4 271.1874 0.61909552 0.27
## 1 277.7514 7.18309340 0.01

GAM are better than GLM, and all GAM AIC are equivalent (deltaAIC<2).


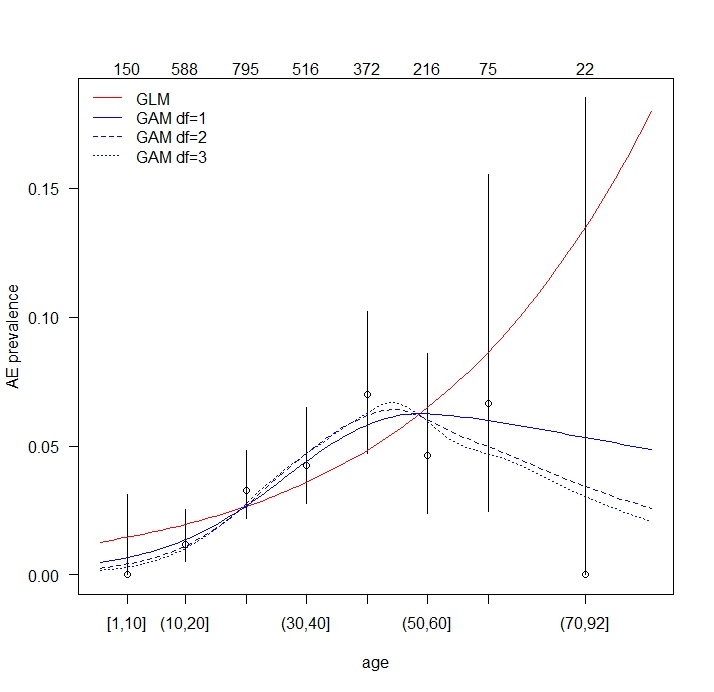


**Visual model comparison**: vertical bars gives 95% confidence interval in age categories; number above the chart is the sample size of each category

After visual examination, considering the small sample size of the oldest category, and for the sake of parsimony, modz2 (GAM df=1) is selected.

summary(modz2)

## Warning in summary.gamlss(modz2): summary: vcov has failed, option qr is used instead

## ******************************************************************
## Family: c("BI", "Binomial")
##
## Call:
## gamlss(formula = aePG ~ cs(age, df = 1), family = BI, data = zhao[,
## c("age", "aePG")])
##
## Fitting method: RS()
##
## ------------------------------------------------------------------
## Mu link function: logit
## Mu Coefficients:
## Estimate Std. Error t value Pr(>|t|)
## (Intercept) -6.62578 0.50770 -13.051 < 2e-16 ***
## cs(age, df = 1) 0.06979 0.01184 5.893 4.32e-09 ***
## ---
## Signif. codes: 0 '***' 0.001 '**' 0.01 '*' 0.05 '.' 0.1 ' ' 1
##
## ------------------------------------------------------------------
## NOTE: Additive smoothing terms exist in the formulas:
## i) Std. Error for smoothers are for the linear effect only.
## ii) Std. Error for the linear terms may not be reliable.
## ------------------------------------------------------------------
## No. of observations in the fit: 2500
## Degrees of Freedom for the fit: 3.000299
## Residual Deg. of Freedom: 2497
## at cycle: 2
##
## Global Deviance: 264.5581
## AIC: 270.5587
## SBC: 288.0326
## ******************************************************************

## 1997-1996 screening

modb1<-gamlss(AECraig~AGE,family=BI,data=barn2[,c("AECraig","AGE")])

## GAMLSS-RS iteration 1: Global Deviance = 809.2691
## GAMLSS-RS iteration 2: Global Deviance = 809.2691

modb2<-gamlss(AECraig~cs(AGE,df=1),family=BI,data=barn2[,c("AECraig","AGE")])

## GAMLSS-RS iteration 1: Global Deviance = 797.8142
## GAMLSS-RS iteration 2: Global Deviance = 797.8141

modb3<-gamlss(AECraig~cs(AGE,df=2),family=BI,data=barn2[,c("AECraig","AGE")])

## GAMLSS-RS iteration 1: Global Deviance = 795.4676
## GAMLSS-RS iteration 2: Global Deviance = 795.4676

modb4<-gamlss(AECraig~cs(AGE,df=3),family=BI,data=barn2[,c("AECraig","AGE")])

## GAMLSS-RS iteration 1: Global Deviance = 794.2953
## GAMLSS-RS iteration 2: Global Deviance = 794.2953

AIC(modb1,modb2,modb3,modb4)

## df AIC
## modb3 3.999550 803.4667
## modb2 2.999569 803.8132
## modb4 4.999376 804.2941
## modb1 2.000000 813.2691

selMod(list(modb1,modb2,modb3,modb4))

## model LL K N2K AIC deltAIC w_i
## 3 cs(AGE, df = 2) -397.7338 3.999550 683.5769 803.4667 0.0000000 0.40
## 2 cs(AGE, df = 1) -398.9070 2.999569 911.4643 803.8132 0.3465111 0.34
## 4 cs(AGE, df = 3) -397.1477 4.999376 546.8682 804.2941 0.8273722 0.26
## 1 AGE -404.6345 2.000000 1367.0000 813.2691 9.8024141 0.00
## AICc deltAICc w_ic
## 3 803.4813 0.0000000 0.40
## 2 803.8220 0.3406457 0.34
## 4 804.3160 0.8347069 0.26
## 1 813.2735 9.7921536 0.00

GAM are better than GLM, and all GAM AIC are equivalent (deltaAIC<2).


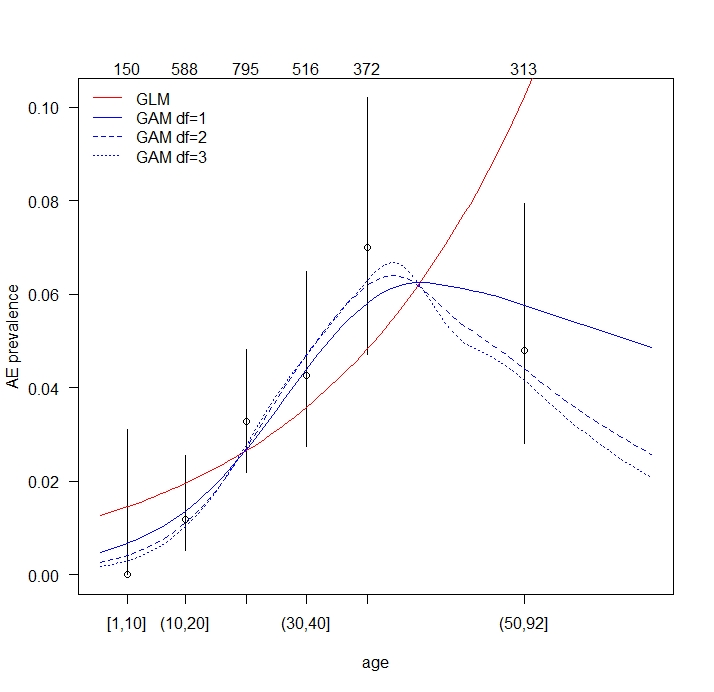


**Visual model comparison**: vertical bars gives 95% confidence interval in age categories; number above the chart is the sample size of each category

After visual examination, modb3 (GAM, df=2) was selected.

summary(modb3)

## Warning in summary.gamlss(modb3): summary: vcov has failed, option qr is used instead

## ******************************************************************
## Family: c("BI", "Binomial")
##
## Call:
## gamlss(formula = AECraig ~ cs(AGE, df = 2), family = BI, data = barn2[,
## c("AECraig", "AGE")])
##
## Fitting method: RS()
##
## ------------------------------------------------------------------
## Mu link function: logit
## Mu Coefficients:
## Estimate Std. Error t value Pr(>|t|)
## (Intercept) -4.643635 0.315623 -14.713 < 2e-16 ***
## cs(AGE, df = 2) 0.038036 0.007833 4.856 1.27e-06 ***
## ---
## Signif. codes: 0 '***' 0.001 '**' 0.01 '*' 0.05 '.' 0.1 ' ' 1
##
## ------------------------------------------------------------------
## NOTE: Additive smoothing terms exist in the formulas:
## i) Std. Error for smoothers are for the linear effect only.
## ii) Std. Error for the linear terms may not be reliable.
## ------------------------------------------------------------------
## No. of observations in the fit: 2734
## Degrees of Freedom for the fit: 3.99955
## Residual Deg. of Freedom: 2730
## at cycle: 2
##
## Global Deviance: 795.4676
## AIC: 803.4667
## SBC: 827.1181
## ******************************************************************
