# Supplementary material for: Long-term retrospective assessment of a transmission hotspot for human alveolar echinococcosis in mid-west China
Source: PLoS Negl Trop Dis. 2019 Aug 30;13(8):e0007701. doi: 10.1371/journal.pntd.0007701 (PMC6742415; doi:10.1371/journal.pntd.0007701)
Supplement: S3 File — (DOC) [file pntd.0007701.s003.doc]

STROBE Statement—checklist of items that should be included in reports of observational studies

|  | Item No | Recommendation |
| --- | --- | --- |
| **Title and abstract** | 1 | (*a*) Indicate the study’s design with a commonly used term in the title or the abstract |
|  |  | (*b*) Provide in the abstract an informative and balanced summary of what was done and what was found  **See Abstract** |
| Introduction | | |
| Background/rationale | 2 | Explain the scientific background and rationale for the investigation being reported  **See 2 introduction** |
| Objectives | 3 | State specific objectives, including any prespecified hypotheses  **See 2 Introduction, last paragraph** |
| Methods | | |
| Study design | 4 | Present key elements of study design early in the paper  **See section 3.1, 3.2 and 3.3** |
| Setting | 5 | Describe the setting, locations, and relevant dates, including periods of recruitment, exposure, follow-up, and data collection  **See section 31, 3.2** |
| Participants | 6 | (*a*) *Cohort study*—Give the eligibility criteria, and the sources and methods of selection of participants. Describe methods of follow-up  **See section 3.2**  *Cross-sectional study*— **See section 3.2** |
|  |  | (*b*) *Cohort study*—For matched studies, give matching criteria and number of exposed and unexposed  **Irrelevant here. As indicated in the MM, patient follow up was done when and where possible through cross-sectional 1994-7, 200-5 screenings and 2014 investigation.** |
| Variables | 7 | Clearly define all outcomes, exposures, predictors, potential confounders, and effect modifiers. Give diagnostic criteria, if applicable  **See section 3.2** |
| Data sources/ measurement | 8* | For each variable of interest, give sources of data and details of methods of assessment (measurement). Describe comparability of assessment methods if there is more than one group  **See section 3.2** |
| Bias | 9 | Describe any efforts to address potential sources of bias  **See section 4.1.2** |
| Study size | 10 | Explain how the study size was arrived at  **See section 3.2.; Study size was dependent on volunteer self-selection and on the coverage of all village.** |
| Quantitative variables | 11 | Explain how quantitative variables were handled in the analyses. If applicable, describe which groupings were chosen and why  **See section 3.2** |
| **See section 3.2**Statistical methods | 12 | (a) Describe all statistical methods, including those used to control for confounding  **See section 3.2 and 4.1.2** |
|  |  | (*b*) Describe any methods used to examine subgroups and interactions  **See section 3.2** |
|  |  | (*c*) Explain how missing data were addressed  **Irrelevant here, but see section 3.2 and 4.1.3** |
|  |  | (*d*) *Cohort study*—If applicable, explain how loss to follow-up was addressed  **See section 3.2 and 4.1.3**  *(e)Cross-sectional study*—If applicable, describe analytical methods taking account of sampling strategy  **See section 3.2 and 4.1.3** |
|  |  | (*e*) Describe any sensitivity analyses  **Irrelevant here.** |
|  |  |  |

Continued on next page

| Results | | |
| --- | --- | --- |
| Participants | 13* | (a) Report numbers of individuals at each stage of study—eg numbers potentially eligible, examined for eligibility, confirmed eligible, included in the study, completing follow-up, and analysed  **See section 4.1.2** |
|  |  | (b) Give reasons for non-participation at each stage  **See section 3.2 and 4.1.2** |
|  |  | (c) Consider use of a flow diagram  **Irrelevant here** |
| Descriptive data | 14* | (a) Give characteristics of study participants (eg demographic, clinical, social) and information on exposures and potential confounders  **See section 4.1** |
|  |  | (b) Indicate number of participants with missing data for each variable of interest  **See section 4.1** |
|  |  | (c) *Cohort study*—Summarise follow-up time (eg, average and total amount)  **See section 4.1** |
| Outcome data | 15* | *Cohort study*—Report numbers of outcome events or summary measures over time  **Irrelevant here** |
|  |  |  |
|  |  | *Cross-sectional study—*Report numbers of outcome events or summary measures  **Irrelevant here** |
| Main results | 16 | *(*a) Give unadjusted estimates and, if applicable, confounder-adjusted estimates and their precision (eg, 95% confidence interval). Make clear which confounders were adjusted for and why they were included  **See Fig. 3, 4 and Table 1** |
|  |  | (*b*) Report category boundaries when continuous variables were categorized  **See table and figures** |
|  |  | (*c*) If relevant, consider translating estimates of relative risk into absolute risk for a meaningful time period  **Not relevant here** |
| Other analyses | 17 | Report other analyses done—eg analyses of subgroups and interactions, and sensitivity analyses  **Irrelevant here** |
| Discussion | | |
| Key results | 18 | Summarise key results with reference to study objectives  Done |
| Limitations | 19 | Discuss limitations of the study, taking into account sources of potential bias or imprecision. Discuss both direction and magnitude of any potential bias  **See discussion and lines 370-383 in particular** |
| Interpretation | 20 | Give a cautious overall interpretation of results considering objectives, limitations, multiplicity of analyses, results from similar studies, and other relevant evidence  **Done, see discussion** |
| Generalisability | 21 | Discuss the generalisability (external validity) of the study results  **Done, see discussion** |
| Other information | | |
| Funding | 22 | Give the source of funding and the role of the funders for the present study and, if applicable, for the original study on which the present article is based  **Done** |

*Give information separately for cases and controls in case-control studies and, if applicable, for exposed and unexposed groups in cohort and cross-sectional studies.

**Note:** An Explanation and Elaboration article discusses each checklist item and gives methodological background and published examples of transparent reporting. The STROBE checklist is best used in conjunction with this article (freely available on the Web sites of PLoS Medicine at http://www.plosmedicine.org/, Annals of Internal Medicine at http://www.annals.org/, and Epidemiology at http://www.epidem.com/). Information on the STROBE Initiative is available at www.strobe-statement.org.
